# Castleman’s disease- a diagnostic dilemma

**DOI:** 10.1186/s13019-014-0170-0

**Published:** 2014-11-28

**Authors:** Anupama Barua, Kostas Vachlas, Richard Milton, James Andrew Charles Thorpe

**Affiliations:** Thoracic Surgery Department, St James’s University Hospital NHS Trust, Beckett Street, Leeds, LS97TF UK

**Keywords:** Benign lung lesion, Castleman’s disease and VATS

## Abstract

Castleman’s disease is a benign lymphoproliferative disease characterised by hyperplasia of lymphoid follicles. It can affect any lymph nodes in the body. Here we describe a caucasian patient who presented with six months history of shortness of breath with CT scan confirming an 8 cm segment of consolidated lung in left hilum. PET scan revealed a mass measuring 68x80x55 mm extending from the left hilum out into the left upper lobe containing area of calcification with SUV max 4.8. The differential diagnosis included atypical sequestration, hamartoma and primary lung malignancy. The patient underwent left video assisted enucleation of the lesion. The histology confirmed the diagnosis of Castleman’s disease.

**Electronic supplementary material:**

The online version of this article (doi:10.1186/s13019-014-0170-0) contains supplementary material, which is available to authorized users.

## Background

Castleman’s disease is known as giant or angiofollicular lymph node hyperplasia, lymphoid hamartoma, or angiofollicular lymph node hyperplasia. It is named after Dr Benjamin Castleman who described this disease in 1954 from Massachusetts General Hospital [1]. The aetiology of the disease is unknown. It can develop in a single lymph node or series of lymph nodes. Castleman’s disease has been described as two clinical identities (i) unicentric/solitary/localised that involves single site for which removal of lymph node is curative and (ii) multicentric or systemic form of Castleman’s disease, which is associated with involvement of multiple lymph nodes and presents with systemic symptoms. Treatment of the systemic form involves corticosteroid; systemic chemotherapy or radiotherapy can be considered for disease control. This report presents a case of hyaline vascular variant of Castleman’s disease which was treated with left video assisted thoracoscopic surgery.

## Case presentation

**Figure 1:**
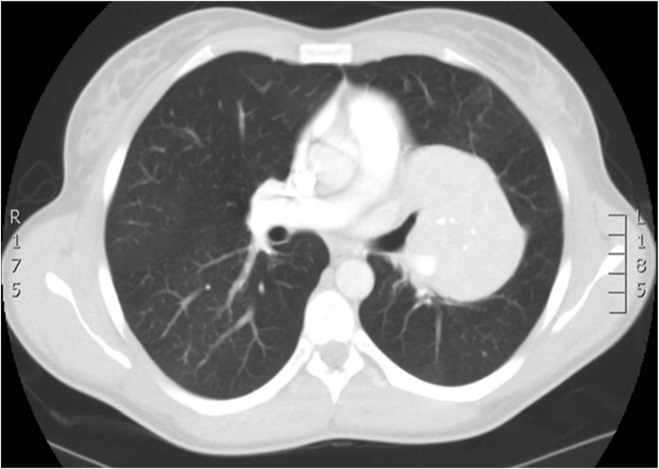
**CT scan of the Castleman’s disease.**

**Figure 2:**
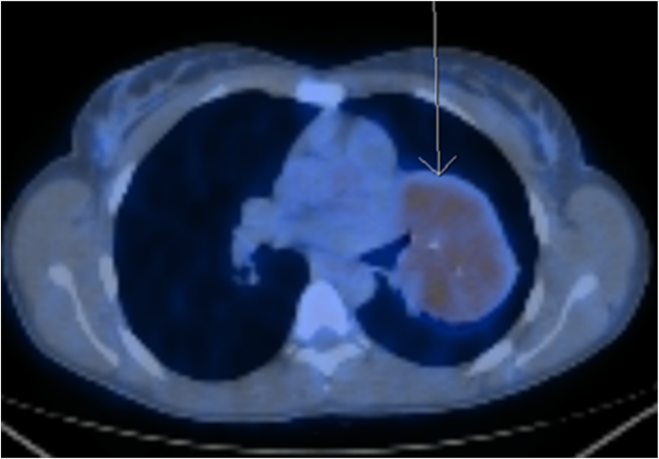
**PET scan of the Castleman’s disease.**

A 24 year old non-smoking caucasian lady presented with six months history of shortness of breath and productive cough, which did not improve with antibiotics. Clinical examination identified a bilateral significant wheeze. Routine haematological and biochemistry tests were normal. Her FEV_1_ was 1.6 l (55% predicted) and FVC 3.04 l (92% predicted). CT revealed an 8 cm well demarcated segment of consolidated lung in the left hilum (Figure 1). On PET scan (Figure 2) it was hypermetabolic returning an SUV max 4.8. The radiological appearance was suggestive of sequestration or hamartoma. A CT-guided biopsy was indeterminate and she was therefore referred for surgery. During VATS, the lesion was identified in the left hilum in a haemorrhagic capsule.

The lesion was partially enucleated; a feeding bronchus, artery and vein were ligated. The histology from the left upper lobe mass revealed hyaline vascular variant of Castleman’s disease. Postoperative recovery was uneventful.

## Discussion

In this case, Castleman’s disease presented as a mass located in the left lung near to the hilum. Attempts at a preoperative diagnosis were unhelpful. CT guided biopsy was inconclusive. She was therefore referred for surgery for diagnostic and therapeutic reasons.

Castleman’s disease may affect anyone from adolescent to seventh decade with equal sex distribution. It is reported to involve any lymph nodes in the body such as cervical (42%), mediastinal (31%), intraabdominal (18%), and retroperitoneal (5%). Only 5% can involve extranodal lymph node [2]. Unicentric Castleman’s disease presents with a slow growing mass, while multicentric variant manifests as fever, malaise, weight loss and generalised lymphadenopathy.

Histologically three types are identified - hyaline vascular variety (90%) and plasma cells type (8-9%) and intermediary mixed type (1-2%) [3]. The hyaline vascular type is identified by dense capillary proliferation and lymphocyte –predominant infiltrate surrounding a small germinal centre. The presence of sheets of mature plasma cells surrounding the normal-large germinal centre is the diagnostic feature of plasma cell variant.

Hyaline variant is generally asymptomatic and may be associated with iron deficiency anaemia and thrombocytopenia whereas the plasma cell verity is associated with infection, lymphoma, immunodeficiency, Kaposi’s sarcoma, non-Hodgkin lymphoma, glomeruloid haemangioma, plasmacytoma, malignancies of colon, kidney and thyroid [4],[5]. POEMS- polyneoropathy, organomegaly, endocrinopathy, monoclonal gammopathy and skin changes are also manifestations of plasma cell type Casleman’s disease [5]. Danon *et al.* suggests that hyline vascular variant may originate from antigen stimulus such as abnormal plasmacytoid monocytes and plasma cell variant could be a response from chronic infection [2].

Diagnosis of localised Castleman’s disease may be difficult in the presence of very few symptoms. On chest radiograph, it may appear as an incidental rounded solitary mediastinal or hilar mass with a differential diagnosis that includes thymoma, lymphoma, neurogenic tumor or bronchial adenoma. In contrast, multicentric Castleman's disease may appear as bilateral hilar and mediastinal enlargement or diffuse reticulonodular pulmonary infiltrations [6].

CT scanning reveals three morphologic patterns of unicentric thoracic Castleman’s disease: a solitary, noninvasive mass (50% of cases); a dominant mass with involvement of contiguous structures (40% of cases); or a matted lymphadenopathy confined to a single mediastinal compartment (10% of cases) [6]. Hypervascularity of the lesion may reveal homogeneously intense contrast enhancement in CT. 5–10% of Castleman's disease demonstrated intralesional calcifications, typically being discrete, coarse, or distinctive with an “arborizing” pattern in enhanced CT [6]. For thoracic Castleman's disease MRI can be used as it demonstrates the extent of the tumour, clarifies its relationship to the bronchovascular structures and shows the feeding vessels. Similar to other inflammatory disease, Castleman’s disease shows mild to moderate FDG uptake in PET scan. Histological diagnosis before surgical removal can be done by CT guided biopsy, EUS and EBUS if the anatomical position permits.

Surgical resection is diagnostic and curative for unicentric Castleman’s disease. As described before, corticosteroid therapy, chemotherapy and monoclonal antibody treatment are suitable for multicentric Castleman’s disease. Surgical excision may not be easy in unicentric hyaline vascular type due to high vascularity. It may be associated with massive haemorrhage at excision and pneumonectomy has been reported for massive bleeding [7]. Embolization of the feeding artery before surgery can be considered to prevent intraoperative bleeding. Complete surgical resection is the gold standard treatment in unicentric Castleman’s disease and is also required for complete recovery in the majority of cases. The prognosis is good after surgical excision in unicentric Castleman’s disease, and 5-year survival is 100% [8]. On the contrary, multicentric Castleman’s disease has a poor prognosis with a median survival of thirty months. Nevertheless, splenectomy with systemic chemotherapy and steroids can improve the prognosis in multicentric Castleman’s disease [9]. Although Castleman’s disease is classed as a benign disease, long term follow up is required as recurrence can happen as late as eight years after the diagnosis of the disease [8].

## Conclusion

This is a rare case report of Castleman's disease. It is important to remember Castleman's disease as a differential diagnosis in benign lung parenchymal lesion. In this case the patient was treated by VATS enucleation of the lesion avoiding unnecessary lung resection. To our knowledge, this is the first lesion which was removed by keyhole surgery.

## Consent

Written informed consent was obtained from the patient for publication. A copy of the written consent is available for review by the Editor-in –Chief of this journal.

## Authors’ original submitted files for images

Below are the links to the authors’ original submitted files for images. Authors’ original file for figure 1 Authors’ original file for figure 2

## Abbreviation

VATS: Video assisted thoracoscopic surgery
