# Supplementary figures and images for: Castleman’s disease- a diagnostic dilemma
Source: J Cardiothorac Surg. 2014 Nov 28;9:170. doi: 10.1186/s13019-014-0170-0 (PMC4255971; doi:10.1186/s13019-014-0170-0)

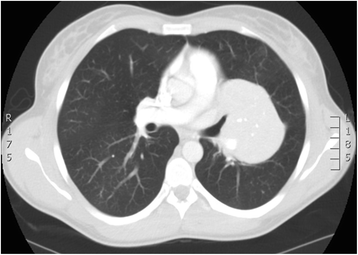

Supplement: Supplementary file 1 — Authors’ original file for figure 1 [file 13019_2014_170_MOESM1_ESM.gif]

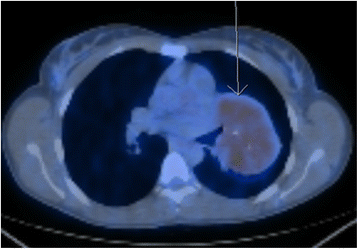

Supplement: Supplementary file 2 — Authors’ original file for figure 2 [file 13019_2014_170_MOESM2_ESM.gif]
